# Perceived distress and its association with depression and anxiety in breast cancer patients

**DOI:** 10.1371/journal.pone.0172975

**Published:** 2017-03-15

**Authors:** Chong Guan Ng, Salina Mohamed, Kiran Kaur, Ahmad Hatim Sulaiman, Nor Zuraida Zainal, Nur Aishah Taib, MyBCC Study group

**Affiliations:** 1 Department of Psychological Medicine, University Malaya Medical Centre, Kuala Lumpur, Malaysia; 2 Department of Psychiatry, Faculty of Medicine, Universiti Teknologi MARA, Kuala Lumpur, Malaysia; 3 Department of Surgery, University Malaya Medical Centre, Kuala Lumpur, Malaysia; Taipei Medical University, TAIWAN

## Abstract

**Background:**

Breast cancer patients often experience a high level of distress. Psychological distress is a broad construct encompass both depression and anxiety. Previous studies in examining which of these psychological symptoms (either anxiety or depression) were more significantly associated with the distress level in breast cancer patients is lacking. This study aims to compare the level of depression and anxiety between patients with different level of distress. The correlation between the changes in distress level with depression or anxiety over 12 months was also examined.

**Methods:**

This study is from the MyBCC cohort study. Two hundred and twenty one female breast cancer patients were included into the study. They were assessed at the time of diagnosis, 6 months and 12 month using Hospital Anxiety and Depression Scale (HADS) and distress thermometer. The information on age, ethnicity, treatment types and staging of cancer were collected.

**Results:**

50.2%, 51.6% and 40.3% of patients had perceived high level of distress at baseline, 6 months and 1 year after diagnosis. Those with high perceived level of distress had significant higher anxiety scores even after adjusted for the underlying depressive scores (Adjusted OR at baseline = 1.28, 95% CI = 1.13–1.44; adjusted OR at 6 months = 1.27, 95% CI = 1.11–1.45; adjusted OR at 12 months = 1.51, 95% CI = 1.29–1.76). There were no significant differences in the depressive scores between the subjects with either low or high distress level. There was reduction in perceived level of distress, anxiety and depression scores at 12 months after the diagnosis. The decrease of distress was positively correlated with the reduction of anxiety scores but not the changes of depressive scores (*r’* = 0.25).

**Conclusion:**

Anxiety is a more significant psychological state that contributed to the feeling of distress in breast cancer as compared with depression. Levels of anxiety at diagnosis in this study would justify screening for anxiety, early identification and therapy for maintaining the psychological well-being of breast cancer patients. Further studies will be needed to measure the effectiveness of therapeutic interventions.

## Introduction

Breast cancer is the most common malignancy in women worldwide [1]. The diagnosis and treatment for breast cancer in women was well known in leading to significant psychological distress [2–5]. The prevalence of psychological distress in cancer patients is reported to be above 30%. [4, 6] Despite its impact on the daily functioning, distress in cancer patients is often overlooked and under-treated [6–8], which possibly lead to poor treatment compliance and survival [9]. In 2003, the National Comprehensive Cancer Network (NCCN) use the word “distress” to describe the psychiatric problem in cancer patients because it is more accepted and less stigmatizing than other psychological or psychiatric term. Distress is defined as “a multifactorial unpleasant emotional experience of a psychological (cognitive, behavioral, emotional), social, and/or spiritual nature that may interfere with the ability to cope effectively with cancer, its physical symptoms, and its treatment” [10]. Psychological distress is broad in construct, covering a wide continuum of emotions and psychiatric symptoms such as depression and anxiety [11].

Anxiety is one of the most common psychological symptoms in breast cancer patients, with the rates ranging from 10 to 30% [12]. It is a state of intense apprehension, uncertainty, and excessive fear as a response to unpleasant stimuli. Anxiety has a multi-dimensional construct which involve cognitive, physiological and physical reactions. Anxiety has been shown to cause fatigue and poor treatment outcome, have impact on the quality of life, and influencing the neuroendocrine and immune systems of the breast cancer patients [13]. Breast cancer patient is overwhelmed with anxiety symptoms due to the anticipation of negative outcomes [14], face extensive uncertainty about the future, concern over recurrence and fear of treatment side effects [15, 16]. Although it is always believed that anxiety was less frequent in women with breast cancer as compared with depression [17], there were findings to indicate that anxiety is more prevalent [18].

Among all types of cancer, the prevalence of depression is the third highest among patients with breast cancer patients after pancreatic and head and neck cancer [19]. This could due to the fact that women with breast cancer are generally reluctant to disclose their depressive symptoms [20]. Another reason could be that physicians are not familiar with the diagnosis of depression in cancer patients [21]. Diagnosis of depression can be challenging in cancer patients due to the overlapping of depressive symptoms with physical symptoms as a consequence of the illness or treatment. Depression affects quality of life, self-care, treatment compliance and outcome of therapy and survival of breast cancer patients [22–25]. It was suggested that other than clinical classical symptomatology of depression such as sadness, anhedonia, guilt, helplessness, hopelessness, suicidal ideation, the other risk factors of depression such as past history of depression, cancer related concerns, lack of confiding relationship and neuroticism personality must be looked at [26, 27]. The rate of depression in breast cancer patients was estimated between 10 to 30% depending on the study population, study design and choice of depression measure. Estimates based on screening instruments were generally higher and more variable than estimates based on structured interviews [23].

Both anxiety and depression have tremendous impact on breast cancer patients. Untreated, both psychological symptoms significantly impact on the patients’ treatment regime, quality of life and may increase their suicide risk. As distress in cancer patients encompasses a spectrum of emotions from normal feelings of sadness and fear to an actual psychiatric disorder, it is difficult to determine if depression or anxiety or both is causing the distress. Previous studies in examining which of these psychological symptoms (either anxiety or depression) were more significantly associated with the distress level in breast cancer patients is lacking. Pandey et al (2007) attempted to answer this by studying 123 head and neck cancer patients. The authors used Distress Inventory for Cancer version 2 (DIC2) and the Hospital Anxiety and Depression scale (HADS) and found that total distress, emotional and social distress subscales were found to have positive correlation with anxiety and depression suggesting a possible overlap of the two constructs [22]. However, it is important to determine the relationship further as psychiatric disorders require a different treatment approach.

In the current study, we aim to investigate the association of anxiety and depression with perceived level of distress among breast cancer patients in Malaysia in one year prospectively.

## Methods

This study is part of a larger study, the Malaysian Breast Cancer Cohort (MyBCC). MyBCC is a prospective cohort study aims to identify the association between genetics, lifestyle and nutrition on overall survival and quality of life of Malaysian breast cancer patients. Please refer to the previous literature for further information of MyBCC cohort study [28]. In the current study, we mainly studied the association of depression and anxiety with perceived distress among the breast cancer subjects at diagnosis, 6 months and 12 months after diagnosis.

The study subjects of the current study were recruited from University Malaya Medical Centre in Kuala Lumpur, Malaysia. The subjects are those who were diagnosed with breast cancer since 1 May 2011. Inclusion criteria were (i) breast cancer that was confirmed by histological examination, (ii) able to complete the necessary interviews and questionnaires, and (iii) able to understand the objective of the study and provide informed consent. Exclusion criteria were (i) secondary breast cancer, (ii) having confusion or delirium, and (iii) male patients. The purpose and details of the study were explained to all potential subjects. The query from the subjects was addressed. Those patients who agreed to participate and given written informed consent were enrolled. Ethical approval was obtained from the Medical Ethic Committee, University Malaya Medical Centre.

### Procedure and measures

The following scales were used for assessment at the time of diagnosis, 6 months and 12 months follow-up visits thereafter. Information on age and ethnicity were obtained. The American Joint Committee on Cancer Staging System for breast cancer was used in this study.

### Hospital anxiety and depression scale

Anxiety and depression were assessed using the Malay Version of Hospital Anxiety and Depression Scale (HADS). HADS was the most frequently reported measure in cancer studies and shown to be the best performing measure for each trajectory stage of the disease. It is a self-administered questionnaire that screened for anxiety (7 items) and depressive (7 items) symptoms. It has demonstrated good reliability. The anxiety (HADS-A) and depression (HADS-D) subscales are scored from 0 to 3 (four-point Likert scales), giving maximum scores of 21 for anxiety and depression respectively [29]. The Malay version of HADS has a good reliability and has been validated among the Malaysian population [30].

### Distress thermometer

The Distress Thermometer is a validated rapid screening tool for psychological distress and has been endorsed by the NCCN Distress Management Guidelines panel. It serves as an initial single item question screen, which identifies distress from any sources. The word ‘‘distress” was chosen because it sounds ‘‘normal” and is less embarrassing to patients. It assesses how much distress patients are going through in the past week. The subjects were instructed to circle from a scale of 0 to 10 to indicate their distress level. “0” means no distress and “10” means extremely distress [31] In the previous literature review, most studies showed that the score of 4 was used as the cut-off score of high distress level with the optimal sensitivity and specificity relative to established criterion [32].

### Statistical analysis

Descriptive statistics for the age, ethnicity and staging of cancer was performed. Study subjects were categorized into moderate to severely distress (distress thermometer score of 4 and above) and low distress (distress thermometer score below 4). The means and standard deviation of HADS-anxiety subscale and HADS-depression subscale scores for both groups were calculated at all three time points (baseline, 6 months and 12 months). The differences between the means of both groups were determined using Independent T test. Logistic regression analysis was further performed to determine the means differences of anxiety scores between the two groups adjusting for the underlying depression scores; or the means differences of the depression scores between the two groups adjusting for the underlying anxiety scores. In the secondary analysis, the changes of distress scores, HADS-anxiety subscale scores and HADS-depression subscale scores at 12 months from baseline were calculated. The correlation between the changes of distress scores with HADS-anxiety scale scores and HADS-depression subscale scores was tested with Spearman’s correlation test. Partial correlation tests were further conducted to examine the correlation between the changes of distress scores with HADS-anxiety subscale scores by adjusting for the changes of HADS-depression scale scores and vice versa. The inernal consistency (Cronbach’s alpha) was calculated for HADS-anxiety and depression subscales for all time points. All tests were two-tailed with significant level of 0.05.

## Results

At the time of data analysis, 221 female subjects with breast cancer were recruited in MyBCC and had completed the 12 months follow up visit. The average age of the subjects was 55 years old (SD = 11.5) with almost half were Chinese, followed by Malay (32.1%) and Indian (17.2%) ethnicity. Twenty six (11.8%) had non-invasive breast cancer (stage 0), 60 (27.1%) had Stage I, 83 (37.6%) had Stage II, 44 (19.9%) had Stage III and 8 (3.6%) had Stage IV disease. Nearly all (95.5%) had surgery with almost two third had chemotherapy (59.7%) and radiotherapy (68.3%). Only about 31% had received hormonal therapy (Table 1).

**Table 1:** Characteristics of the subjects (N = 221).

Characteristic	
Age, mean (sd)	55.13 (11.5)
Ethnicity, n (%)	
Malay	71 (32.1)
Chinese	108 (48.9)
Indian	38 (17.2)
Others	2 (0.9)
Staging	
0	26 (11.8)
I	60 (27.1)
II	83 (37.6)
III	44 (19.9)
IV	8 (3.6)
Surgery, n (%)	211 (95.5)
Chemotherapy, n (%)	132 (59.7)
Radiotherapy, n (%)	151 (68.3)
Hormonal therapy, n (%)	69 (31.2)

Overall the mean scores for anxiety and depression were low at each time points (Table 2). 50.2%, 51.6% and 40.3% of patients had perceived high level of distress at baseline, 6 months and 1 year after diagnosis. Breast cancer patients with high perceived level of distress had higher anxiety scores even after adjusted for the underlying depressive scores at all three time points (Adjusted OR at baseline = 1.28, 95% CI = 1.13–1.44; adjusted OR at 6 month = 1.27, 95% CI = 1.11–1.45; adjusted OR at 12 months = 1.51, 95% CI = 1.29–1.76). There were no significant differences in the depressive scores between the two groups after adjusting for the underlying anxiety scores (Table 2).

**Table 2:** The comparison of anxiety and depression scores between breast cancer patients with high or low perceived level of distress (N = 211).

Time point: Baseline					
	Distress score, mean (sd)		Adjusted OR	95% CI	*p*
	4 and above	Less than 4			
Anxiety score	6.76 (4.01)	4.06 (2.72)	1.28	1.13–1.44	< 0.01
Depression score	4.75 (3.92)	3.05 (2.64)	1.00	0.89–1.12	0.96
Time point: 6 months					
	Distress score, mean (sd)		Adjusted OR	95% CI	*p*
	4 and above	Less than 4			
Anxiety score	5.96 (3.25)	3.38 (3.09)	1.27	1.11–1.45	< 0.01
Depression score	4.99 (3.62)	3.14 (2.85)	1.04	0.91–1.18	0.60
Time point: 1 year					
	Distress score, mean (sd)		Adjusted OR	95% CI	*p*
	4 and above	Less than 4			
Anxiety score	5.44 (3.18)	2.63 (2.50)	1.51	1.29–1.76	< 0.01
Depression score	4.55 (2.98)	3.06 (2.93)	0.92	0.80–1.06	0.24

CI = confidence interval, OR = odds ratio, distress = perceived level of distress measured with distress thermometer, anxiety = scores of hospital anxiety and depression scale (HADS)—anxiety subscale, depression = scores of HADS—depression subscale scores.

There was reduction in perceived level distress scores, anxiety scores and depression scores at 12 months as compared to the baseline. The change of anxiety scores was positively correlated with the change of distress scores even after adjusted for the changes of depression scores (*r’* = 0.25). There was no significant correlation between the changes of depression scores with the changes of distress scores (Table 3). The internal consistency for HADS-anxiety subscales items ranged from 0.77 to 0.80 for all three time points. For the HADS-depression subscales items, the Cronbach’s alpha ranged from 0.68 to 0.69 for all three time points (Table 3).

**Table 3:** The correlation between changes in perceived distress with the changes in anxiety and depression scores among the breast cancer patients (N = 211).

	Baseline	12 months	Mean difference	95% CI	*p* value	*r'*
Distress,mean (SD)	3.55 (2.74)	3.06 (2.39)	0.49	0.06, 0.91	0.02	-
Anxiety,mean (SD)	5.42 (3.69)	3.78 (3.11)	1.16	1.10, 2.18	<0.01	0.25*
Depression,mean (SD)	3.90 (3.45)	3.60 (3.08)	0.30	-0.27, 0.87	0.03	-0.02

CI = confidence interval, *r’* = partial correlation with changes of distress after adjusting for the changes of either anxiety or depression scores, distress = perceived level of distress measured with distress thermometer, anxiety = scores of hospital anxiety and depression scale (HADS)—anxiety subscale, depression = scores of HADS—depression subscale scores,

*significance at the level of 0.01

## Discussion

In the current study, we demonstrated that perceived distress among the breast cancer patients was significantly associated with anxiety but not depression. This finding was reflected at each point of the study—at baseline, 6 months and 12 months; where patients who were moderate to severely distress scored higher in the anxiety scale. There was no difference in the depression scores regardless of the distress level of the breast cancer patients. The association was further shown in the positive correlation between the changes of distress with the changes of anxiety scores. Over the 12 months period, the reduction of distress was associated with the reduction of anxiety. In contrast, reduction of distress had no association with the changes in the depression scores.

The diagnosis and treatment of breast cancer causes a significant impact on the psychological well—being of the patients [4, 5]. However, individual breast cancer patients differ in the extent of their psychological reaction to the illness. Psychological distress is a broad construct, covering a wide continuum of symptoms ranging from common normal feeling of vulnerability and fear to mental disabling conditions such as depression, anxiety and adjustment disorder [1, 11, 31]. The commonest psychological states associated with distress in breast cancer patients are anxiety and depression [33, 34]. In our study, 50% of the subjects experienced high level of distress. The result is similar To previous study reported that up to 50% of the women with breast cancer experience high levels of distress with more than 30% of the women with early breast cancer had depression, anxiety, or both at diagnosis [35]. Many studies reported that depression was more common in breast cancer patients. Depressed breast cancer patients were less proactive in seeking treatments, have more severe symptoms, poorer response to systemic therapy, longer recovery times and poor outcomes [36–38]. Some other studies argued that anxiety is a more dominant psychological problem in breast cancer patients. Patients with breast cancer face extensive uncertainty about the disease progression, concern over potential recurrence, and fear of physical suffering [39]. All of these feelings contribute to the elevated anxiety levels. Either depression or anxiety has remarkably deleterious effects on the breast cancer patients’ quality of life [40].

It always believed that anxiety is secondary as compared to depression in the spectrum of psychological distress among breast cancer patients [17]. However, we demonstrated that the perceived level of distress among the breast cancer patients was positively associated with the level of anxiety but not depression in the current study. It was also shown in other previous studies that anxiety prevails throughout the period of treatment and recovery for patients with breast cancer, even among the disease-free breast cancer survivors [39, 41]. Anxiety occurs when the adaptive response to the diagnosis or treatment of cancer is excessive and impairing the ability to cope with stress. Therefore, among breast cancer patients, uncertainty plays an important role affecting the illness experience, adaptation, and has a detrimental effect on the physical, mental and sexual quality of life [35].

Breast cancer patients who anticipate more negative effects from the disease and treatment in the beginning experience a higher level of anxiety [42]. We have shown that the level of distress and anxiety was higher at the diagnosis and significantly reduced over a 12 months period. The reduction of distress was also significantly correlated with the decrease of anxiety in the current study. This could be explained by conceptualizing anxiety as an anticipatory mood state of upcoming negative events [43]. At the early stage, breast cancer patients have heightened sense of vulnerability and high level of anxiety as they face extensive uncertainty about the diagnosis, progress of cancer, effectiveness and outcome of a treatment [39]. They may have concerns over the possibility of metastasis and fear of treatment side effects. Over a period of time, the breast cancer patients will gradually be coming to terms with the disease and the procedures of the treatment. It renders a progressive decline in the sense of future vulnerability and uncertainty, and consequently, reduces the level of anxiety over time [44]. Uncertainty can be seen as a source of anxiety that lead to psychological distress for breast cancer patients [39]. A previous study showed that women with breast cancer were severely anxious during the diagnostic period [45–47]. Their anxiety levels before diagnosis were significantly higher than after diagnosis, with the highest level before breast biopsy. The authors concluded that breast cancer patients who chronically feeling anxious were more distressed, compared with those who did not generally feel anxious [45–47]. Their results were similar to the current findings where increasing psychological distress was related to higher level of anxiety. It suggests that a high level of anxiety trait as a personality characteristic could be a key factor in psychological adjustment to cancer. It also reflects the vulnerability to stress for people with chronically high levels of anxiety [48].

It is commonly believed that co-morbid depression significantly increases the burden of distress and dysfunction for patients with breast cancer [23]. However, we did not show any association between the levels of depression with perceived distress in the breast cancer patients in the current study. Our results also indicated that there was reduction of depression a year after the cancer diagnosis but it was not correlated with the reduction of distress among the study subjects. This could be explained by the fact that depression in cancer patients is complex and encompasses several aspects. Depression includes a variety of mood disturbances and clinical presentations. Besides the classical depressive symptoms such as low mood, low energy, poor concentration, loss of interests, low self-esteem, guilt feelings, sleep and appetite disturbances and hopelessness, there are features often underrepresented in endogenous depression, such as somatic complaints, psychic and somatic anxiety. Atypical symptoms such as anger, irritability, and hostility were also often neglected in the assessment of depression [49]. This gap often leads to the under-diagnosis of depression in breast cancer.

There were some limitations of the study. First, the study subjects were recruited from a single centre which is a teaching hospital at the capital of Malaysia. The generalizability of the samples could be improved in the future by recruiting patients from different sittings and regions of the country. However, the MyBCC cohort study remains a detailed collection of psychological measures in a cohort study has not been reported in the Southeast Asian country. Second, the other associated factors for distress were not included in the current study such as pain, financial support, family background, physical suffering and social support. Third, we only studied the association for the first 12 months after diagnosis. For future analysis, the association between distress with anxiety and depression could be studied for later period of the disease and on a longer follow-up further analysis to measure the effect of psychological and lifestyle factors on prognosis of patients. Fourth, distress thermometer may have low discriminant validity. It may not be sensitive for the anxiety and depression symptoms. Other recommended assessment tools such as Patient Health Questionnaire 9 (PHQ-9) or Generalized Anxiety Disorder 7 Items Scale (GAD-7) may serve as a more sensitive tool in this context. Lastly, the psychological distress scoring scale used in the study was a single item scale. There are many cancer-specific stresses that may not be detected leading loss of valuable risk factors for psychological distress.

In conclusion, breast cancer patients experienced high level of distress which including depression and anxiety after diagnosis. Over time, the level of distress gradually reduced. Although both anxiety and depression were both commonly found in the breast cancer patients, they differ in their impact on the level of distress. Anxiety which could be related to the feeling of uncertainty seems to play a more significant role in the feeling of distress among the breast cancer patients. In contrast, depression which consists of negative emotions had no association with the level of perceived distress in cancer patients. As compared to depression, anxiety was often neglected in the management of psychological well-being among the breast cancer patients. With the current findings, more focus in helping breast cancer patients in relieving their feeling of anxiety in the future is important in reducing their perceived level of distress.
